# Clinical and epigenetic features of colorectal cancer patients with somatic *POLE* proofreading mutations

**DOI:** 10.1186/s13148-021-01104-7

**Published:** 2021-05-25

**Authors:** Takashi Kawai, Akihiro Nyuya, Yoshiko Mori, Takehiro Tanaka, Hiroaki Tanioka, Kazuya Yasui, Toshiaki Toshima, Fumitaka Taniguchi, Kunitoshi Shigeyasu, Yuzo Umeda, Toshiyoshi Fujiwara, Makoto Okawaki, Yoshiyuki Yamaguchi, Ajay Goel, Takeshi Nagasaka

**Affiliations:** 1 Department of Gastroenterological Surgery, Okayama University Graduate School of Medicine, Dentistry and Pharmaceutical Sciences, Okayama, Japan; 2 Department of Clinical Oncology, Kawasaki Medical School, 577 Matsushima, Kurashiki, 701-0192 Japan; 3 Department of Clinical Genetics and Digestive Tract and General Surgery, Saitama Medical Center, Saitama Medical University, Kawagoe, Saitama 350-8550 Japan; 4 Department of Pathology, Okayama University Graduate School of Medicine, Dentistry and Pharmaceutical Sciences, Okayama, Japan; 5 Department of Molecular Diagnostics and Experimental Therapeutics, Beckman Research Institute of City of Hope Comprehensive Cancer Center, Duarte, CA 91016 USA

**Keywords:** *POLE*, Colorectal cancer, Methylation, Microsatellite instability, *MLH1*

## Abstract

**Background:**

Mutations in the *POLE* gene result in an ultra-hypermutated phenotype in colorectal cancer (CRC); however, the molecular characterisation of epigenetic alterations remains unclear. We examined the genetic and epigenetic profiles of *POLE*-mutant CRC to elucidate the clinicopathological features of the associated genetic and epigenetic alterations.

**Results:**

Tumour tissues (1,013) obtained from a cohort of patients with CRC were analysed to determine associations between the proofreading domain mutations of *POLE* with various clinicopathological variables, microsatellite instability (MSI) status, *BRAF* and *KRAS* mutations, and the methylation status of key regions of *MLH1*,* MGMT*, and *SFRP2* promoters by calculating the methylation scores (range 0–6). Only four cases (0.4%) exhibited pathogenic *POLE* hotspot mutations (two p.P286R [c.857C > G], one p.V411L [c.1231G > C], and p.S459F [c.1376C > T] each), which were mutually exclusive to *BRAF* and *KRAS* mutations and MSI. CRC patients were divided into four subgroups: patients with *POLE* mutations (POLE, 0.4%, *n* = 4), patients with both MSI and extensive methylation in *MLH1* (MSI-M, 2.9%, *n* = 29), patients with MSI but no extensive methylation in *MLH1* (MSI-U, 3.6%, *n* = 36), and patients without MSI (non-MSI, 93.2%, *n* = 944). The POLE group was younger at diagnosis (median 52 years, *P* < 0.0001), with frequent right-sided tumour localisation (frequency of tumours located in the right colon was 100%, 93.1%, 36.1%, and 29.9% in POLE, MSI-M, MSI-U, and non-MSI, respectively; *P* < 0.0001), and was diagnosed at an earlier stage (frequency of stages I–II was 100%, 72.4%, 77.8%, and 46.6% in POLE, MSI-M, MSI-U, and non-MSI, respectively, *P* < 0.0001). The mean methylation score in POLE was not different from that in MSI-U and non-MSI, but the methylation signature was distinct from that of the other subgroups. Additionally, although the examined number of *POLE*-mutant tumours was small, the number of CD8-positive cells increased in tumours with partial methylation in the *MLH1* gene.

**Conclusions:**

CRC patients with *POLE* proofreading mutations are rare. Such mutations are observed in younger individuals, and tumours are primarily located in the right colon. Diagnosis occurs at an earlier stage, and distinct epigenetic alterations may be associated with CD8 cell infiltration.

**Supplementary Information:**

The online version contains supplementary material available at 10.1186/s13148-021-01104-7.

## Background

Most colorectal cancers (CRCs) occur sporadically and progress through sequential accumulation of multiple genetic and epigenetic alterations by influencing the expression and behaviour of genes that regulate cell growth and differentiation [1–3]. Several crucial gene defects in sporadic CRC have been identified, and specific molecular phenotypes have been described, including chromosomal instability (CIN), microsatellite instability (MSI), and the CpG island methylator phenotype (CIMP) [1, 3–5]. Although somatic mutations in the *POLE* gene are found in 3–7% of CRCs, mutations within the proofreading (exonuclease) domain of *POLE* are present in only 1–2% of CRCs [6–9]. The proofreading potential of *POLE* is essential for ensuring replication fidelity, and its disruption by the pathogenic heterozygous mutations found in cancers leads to an ultra-hypermutated phenotype of tumours, with the highest burden of single-nucleotide variants among human cancers [6, 10, 11]. Analogous to CRC, patients with endometrial cancers harbouring pathogenic *POLE* exonuclease domain mutations have excellent prognosis [6, 7, 10, 12], possibly because such an extreme hypermutation event causes the enrichment of antigenic neoepitopes, which in turn stimulates a potent cytotoxic T-cell response in cancer cells [9, 13, 14].

To date, several pivotal studies have characterised *POLE*-mutant CRCs with their corresponding MSI status, genetic mutations, tumour lymphocyte infiltration, and clinicopathological findings, including clinical outcomes [6–8, 15]. However, to the best of our knowledge, no study has thus far characterised *POLE*-mutant CRCs in the context of epigenetic alterations. The present study aimed to evaluate the epigenetic profiles of *POLE*-mutant CRC and elucidate the clinicopathological features associated with key genetic and epigenetic alterations in this malignancy.

## Results

### Detection of pathogenic somatic *POLE* mutations

**Fig. 1:**
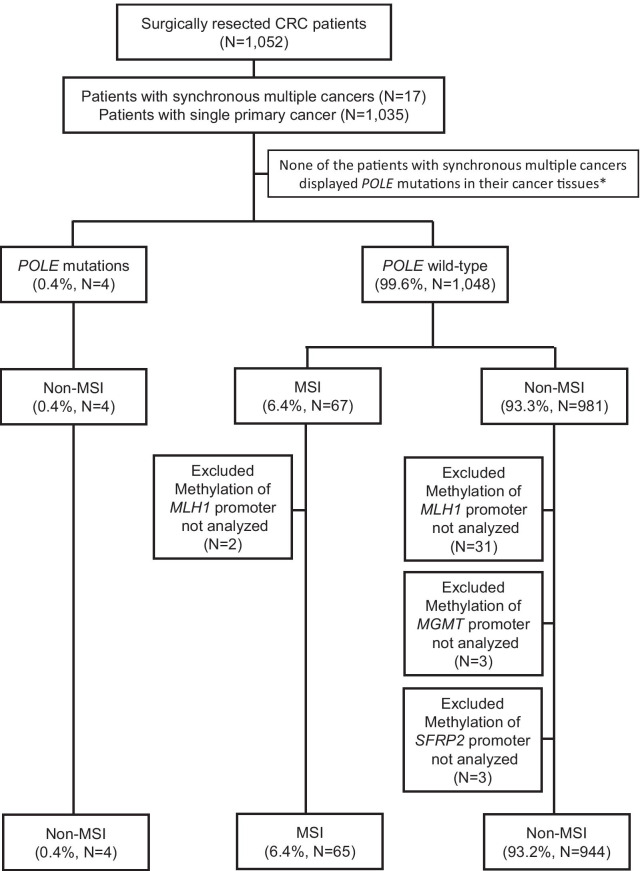
The STROBE diagram of the colorectal cancer patient cohort. *Patients with synchronous multiple cancers based on the most advanced tumour lesion were subsequently confirmed pathologically for further molecular studies

**Fig. 2:**
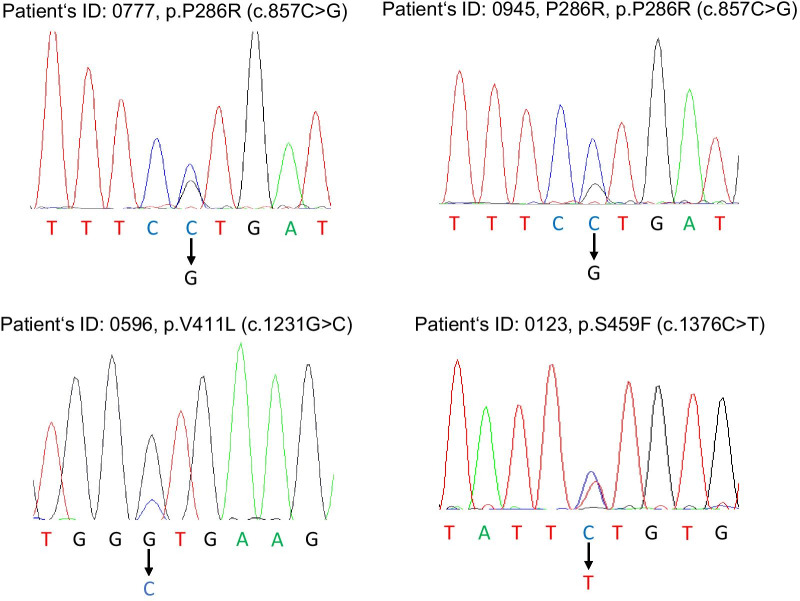
Detection of *POLE* proofreading mutations. Among 1,070 CRC tissues analysed, pathogenic somatic *POLE* proofreading mutations were detected in four CRCs (two cases were p.P286R [c.857C > G], one was p.V411L [c.1231G > C], and one was p.S459F [c.1376C > T]) and DNA from their corresponding normal mucosa displayed no germline mutations

Of the 1,052 CRC patients, 17 had resected synchronous multiple cancers (one patient had three synchronous tumours and 16 patients had double synchronous tumours). Therefore, a total of 1,070 CRC tissues were analysed for *POLE* mutations. None of the patients with synchronous multiple cancers displayed *POLE* mutations in their cancer tissues; thus, patients with synchronous multiple cancers were analysed by the most advanced tumour lesion for further molecular studies (Fig. 1). Pathogenic proofreading *POLE* mutations were detected in four CRCs as recurrent variants and are known to cause an ultra-mutator phenotype and characteristic mutation spectrum (NM_006231.3: two cases were p.P286R [c.857C > G], one was p.V411L [c.1231G > C], and one was p.S459F [c.1376C > T], Fig. 2). Regarding germline mutations, the DNA of four patients was sequenced to determine whether the *POLE* mutations existed in the DNA extracted from their corresponding normal mucosa; however, no germline mutations were present.

### Evaluation of MSI status

The MSI status of the 1,052 CRC tissues was evaluated. None of the four tumours harbouring pathogenic *POLE* mutations displayed MSI features in the four mononucleotide repeat markers [12, 16]. Among the remaining 1,048 CRCs, 6.4% (67/1,048) displayed MSI features, while 93.3% (981/1,048) did not exhibit any MSI features and were deemed microsatellite stable (non-MSI) (Fig. 1).

### Immunohistochemical (IHC) analysis of MMR expression in CRCs with MSI

The expression status of the four DNA mismatch repair (MMR) proteins (MLH1, MSH2, PMS2, and MSH6) was confirmed in all 67 MSI CRC tissues. By IHC analysis, 11.9% (8/67) were classified as MMR-proficient (pMMR), and the remaining 86.8% of MSI CRCs (59/67) were confirmed to be MMR-deficient (dMMR). Of the 59 dMMR tumours, 79.7% (47/59) exhibited both MLH1 and PMS2 deficiency (dMLH1), 15.3% (9/59) exhibited both MSH2 and MSH6-deficiency (dMSH2), 5.1% (3/59) exhibited MSH6-deficiency alone (dMSH6), and none of the tumours displayed PMS2-deficiency on their own (dPMS2). The precise status of IHC staining and MSI markers in MSI tumours is shown in Additional file 1: Table 1.

### Classification of four CRC subtypes according to *POLE* mutations, MSI, and methylation status in the* MLH1* promoter region

**Fig. 3:**
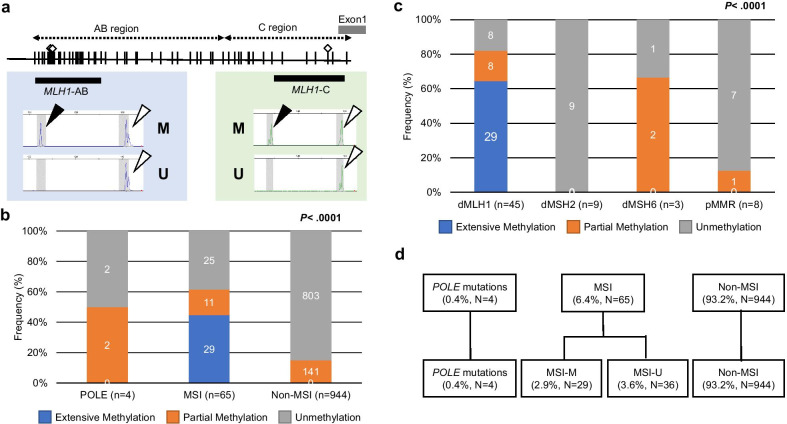
Methylation analysis of the promoter region in the *MLH1* gene. **a** Schematic depiction of two regions (AB and C region) of the *MLH1* promoter for methylation, and results of a panel of representative fluorescent bisulphite PCR following restriction enzyme analysis. AB and C region in this study link to A plus B and C plus D region, defined by Deng and colleagues [18], respectivily.  Methylated samples had the new fragment cleaved by the restriction enzyme (black triangles). White triangles represent unmethylated alleles. M and U denote methylated and unmethylated, respectively. A grey bar denotes an untranslated exon. **b** The frequencies of *MLH1* promoter methylation status among the POLE, MSI, and non-MSI groups. Extensive methylation is defined as 5.0% or more methylation on both AB and C regions. Partial methylation is defined as 5.0% or more methylation on AB region only. **c** The frequencies of *MLH1* promoter methylation status according to MMR protein expression status. dMLH1, dMSH2, and dMSH6 denote deficiency of MLH1, MSH2, and MSH6, respectively. pMMR denotes proficiency of all MMR proteins. **d** The final classification of 1,013 CRCs according to *POLE* mutation, MSI, and *MLH1* promoter methylation status. MSI-M denotes MSI tumours with extensive *MLH1* methylation. MSI-U denotes MSI tumours without extensive *MLH1* methylation. The number in each column denotes the number of samples classified in each category divided by *MLH1* methylation status

Sporadic MSI tumours are primarily caused by inactivation of the *MLH1* gene, which has a large CpG island within its promoter region that divides it into at least two discrete regions of methylation (the AB and C regions in this study, Fig. 3a) [17–20]. Inactivation of *MLH1* was observed when CpG methylation spread through the AB to the C region.

In this cohort, no methylation in the AB or C region (unmethylated) was observed in 50.0% (2/4) of *POLE*-mutant CRCs, 38.5% (25/65) of MSI CRCs, and 85.1% (803/944) of non-MSI CRCs. Partial methylation in *MLH1* (i.e. affecting the AB region only) was observed in 50.0% (2/4) of *POLE*-mutant CRCs, 16.9% (11/65) of MSI CRCs, and 14.9% (141/944) of non-MSI CRCs. Extensive methylation (i.e. affecting both the AB and C regions) was observed in 44.6% (29/65) of MSI CRCs (*P* < 0.0001, Fig. 3b) and 64.4% (29/45) of dMLH1 tumours, which were suspected to be sporadic MSI tumours. In contrast, no extensive methylation was observed in either dMSH2 or dMSH6 cancers, including in patients with Lynch syndrome (Fig. 3c). Therefore, in this study, we classified MSI tumours according to their methylation status in the *MLH1* promoter; 2.9% (29/1,013) tumours with extensive *MLH1* methylation were categorised as MSI with *MLH1* methylation (MSI-M) and 3.6% (36/1,013) tumours without extensive *MLH1* methylation as MSI with unmethylated *MLH1* (MSI-U).

Finally, we classified 1,013 CRC patients into four subgroups: non-MSI CRC patients with *POLE* mutations (*POLE* group, 0.4%, *n* = 4), MSI CRC patients with extensive *MLH1* methylation (MSI-M group, 2.9%, *n* = 29), MSI CRC patients with unmethylated *MLH1* (MSI-U group, 3.6%, *N* = 36), and non-MSI CRC patients (non-MSI group, 93.2%, *n* = 944, Fig. 3d).

### Clinical and genetic features of CRC patients with *POLE* mutations

Table 1 illustrates in detail associations between the four CRC subgroups and their clinical and genetic features. We observed that a significant proportion of tumours classified within the POLE group occurred in younger patients with a mean age of 52.5 years. Additionally, all POLE (4/4) and 93.1% (27/29) of MSI-M were proximally located, in contrast to 36.1% (13/36) of MSI-U and 29.9% (282/944) of non-MSI. All patients with *POLE* mutations were diagnosed at an earlier stage (4 out of 4 at stages I–II), similar to 72.4% (21/29) of MSI-M and 77.8% (28/36) MSI-U, compared to 46.6% (440/944) of non-MSI (*P* < 0.0001).

**Table 1:** Association between the clinicopathological features of CRC patients stratified by *POLE* mutation, MSI, and *MLH1* methylation status

Characteristics		POLE (*n* = 4)	MSI-M (*n* = 29)	MSI-U (*n* = 36)	non-MSI (*n* = 944)	*P* value
Age	Mean (range)	52.5 (41–63)	76.9 (60–89)	59.5 (24–87)	66.0 (21–95)	< .0001
	> 70	0 (0)	22 (75.9)	7 (19.4)	365 (38.7)	< .0001
	55–69	2 (50%)	7 (24.1)	18 (50.0)	441 (46.7)	
	40–54	2 (50%)	0 (0)	7 (19.4)	118 (12.5)	
	< 39	0 (0)	0 (0)	4 (11.1)	20 (2.1)	
Gender	Female	2 (50%)	19 (65.5%)	13 (36.1%)	389 (41.2%)	0.0593
	Male	2 (50%)	10 (34.5%)	23 (63.9%)	555 (58.8%)	
Tumour location	Right	4 (100%)	27 (93.1%)	13 (36.1%)	282 (29.9%)	< .0001
	Left	0 (0)	2 (6.9%)	23 (63.9%)	662 (70.1%)	
Histology	Well	0 (0)	7 (24.1%)	6 (16.7%)	276 (29.2%)	< .0001
	Moderate	3 (75%)	7 (24.1%)	20 (55.6%)	571 (60.5%)	
	Poor/muc	1 (25%)	15 (51.7%)	10 (27.8%)	97 (10.3%)	
UICC stage	I	1 (25%)	11 (37.9%)	14 (38.9%)	197 (20.9%)	0.0011
	II	3 (75%)	10 (34.5%)	14 (38.9%)	246 (26.1%)	< .0001*
	III	0 (0)	6 (20.7%)	4 (11.1%)	297 (31.5%)	
	IV	0 (0)	2 (6.9%)	4 (11.1%)	204 (21.6%)	
*RAS* mutational status	*BRAF* mutation	0 (0)	20 (69.0%)	4 (11.1%)	35 (3.7%)	< .0001
	*KRAS* mutation	0 (0)	0 (0)	8 (22.2%)	307 (32.5%)	
	Wild-type	4 (100%)	9 (31.0%)	24 (66.7%)	602 (63.8%)	
Methylation score	Mean (range)	2 (2)	3.2 (2–4)	1.9 (0–4)	1.9 (0–4)	< .0001
*MGMT* methylation	Extensive	0 (0)	17 (58.6%)	4 (11.1%)	166 (17.6%)	< .0001
	Partial	0 (0)	1 (3.5%)	5 (13.9%)	60 (6.4%)	
	Unmethylation	4 (100%)	11 (37.9%)	27 (75.0%)	718 (76.1%)	
*SFRP2* methylation	Extensive	4 (100%)	28 (96.6%)	23 (63.9%)	602 (63.8%)	0.0158
	Partial	0 (0)	1 (3.5%)	8 (22.2%)	194 (20.6%)	
	Unmethylation	0 (0)	0 (0)	5 (13.9%)	148 (15.7%)	

*P-values* were calculated using the Chi-squared test

^*^ *P-*value was calculated between stages I–II and III–IV

*BRAF* and *KRAS* mutations were not observed in *POLE*-mutant tumours. In contrast, *BRAF* mutations were observed in 69.0% (20/29) of MSI-M, 11.1% (4/36) of MSI-U, and 3.7% (35/944) of non-MSI, whereas *KRAS* mutations were observed in 22.2% (8/36) of MSI-U and 32.5% (307/944) of non-MSI (*P* < 0.0001).

### Epigenetic features of CRC patients with *POLE* mutations

To better understand the differences between tumours with *POLE* mutations and the other subtypes, we evaluated the methylation status of discrete regions within the promoter of the *MGMT* and *SFRP2* genes.

**Fig. 4:**
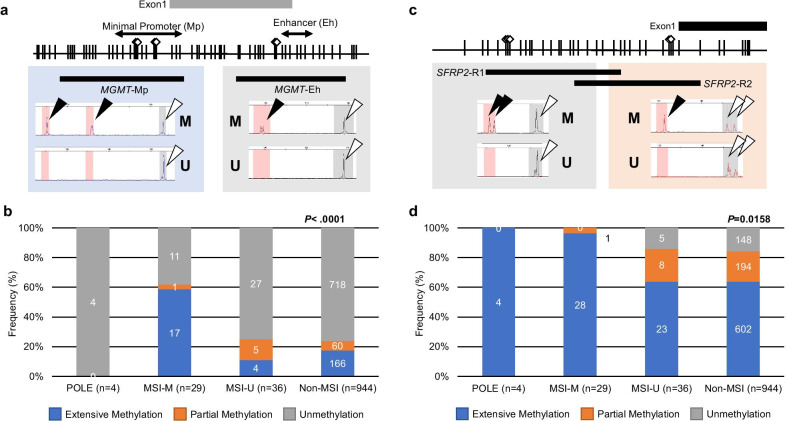
Methylation analysis of the promoter region in the *MGMT* and *SFRP2* gene. **a** Schematic depiction of two regions (Mp and Eh region) of the *MGMT* promoter for methylation, and results of a panel of representative fluorescent bisulphite PCR following restriction enzyme analysis. Methylated samples had the new fragment cleaved by the restriction enzyme (black triangles). White triangles represent unmethylated alleles. M and U denote methylated and unmethylated, respectively. A grey bar denotes an untranslated exon. **b** The frequencies of *MGMT* promoter methylation status among the POLE, MSI-M, MSI-U, and non-MSI groups. Extensive methylation is defined as 5.0% or more methylation on both Mp and Eh regions. Partial methylation is defined as 5.0% or more methylation on either Eh or Mp region. **c** Schematic depiction of two regions (R1 and R2 region) of the *SFRP2* promoter for methylation, and results of a panel of representative fluorescent bisulphite PCR following restriction enzyme analysis. Methylated samples had the new fragment cleaved by the restriction enzyme (black triangles). White triangles represent unmethylated alleles. M and U denote methylated and unmethylated, respectively. A black bar denotes translated exon. **d** The frequencies of *SFRP2* promoter methylation status among the POLE, MSI-M, MSI-U, and non-MSI groups. Extensive methylation is defined as 5.0% or more methylation on both R1 and R2 region. Partial methylation is defined as 5.0% or more methylation on either R1 or R2 region. The number in each column denotes the number of samples classified in each category divided by *MGMT* or *SFRP2* methylation status

Concerning *MGMT* methylation status, lack of methylation in the minimal promoter (Mp) and enhancer (Eh) regions (defined as unmethylated) was observed in 100% (4/4) of POLE, 37.9% (11/29) of MSI-M, 75.0% (27/36) of MSI-U, and 76.1% (718/944) of non-MSI. Partial methylation in *MGMT* (i.e. affecting either MP or Eh) was observed in none of the POLE cases, but in 3.5% (1/29) of MSI-M, 13.9% (5/36) of MSI-U, and 6.4% (60/944) of patients without MSI. Extensive methylation of *MGMT* (i.e. affecting both Mp and Eh) was observed in none of the POLE, 58.6% (17/29) of MSI-M, 11.1% (4/36) MSI-U, and 17.6% (166/944) non-MSI (*P* < 0.0001, Fig. 4a, b).

In contrast to *MGMT* methylation status, lack of methylation in both region-1 (R1) and region-2 (R2) in *SFRP2* (defined as unmethylated) was not observed in POLE or MSI-M, but in 13.9% (5/36) of MSI-U and 15.7% (148/944) of non-MSI. Partial methylation in *SFRP2* (i.e. affecting either R1 or R2) was observed in none of the POLE, but in 3.5% (1/29) of MSI-M, 22.2% (8/36) of MSI-U, and 20.6% (194 of 944) of non-MSI patients. Extensive methylation of *SFRP2* (i.e. affecting both R1 and R2) was observed in all of the POLE, 96.6% (28/29) of MSI-M, 63.9% (23/36) of MSI-U, and 63.8% (602/944) of non-MSI (*P* = 0.0158, Fig. 4c, d).

**Fig. 5:**
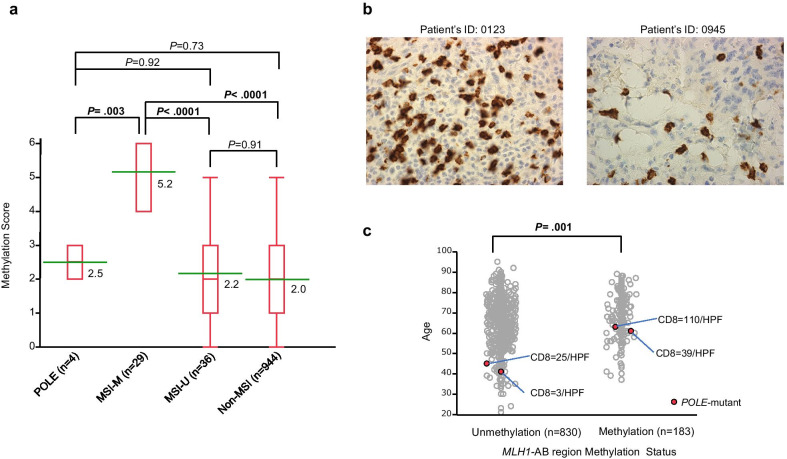
Features of *POLE*-mutant CRCs. **a** Mean methylation score in various subgroups of CRCs categorised by *POLE* mutation, MSI, and *MLH1* promoter methylation status. The mean methylation score in each subset was calculated based on the *MLH1*, *MGMT*, and *SFRP2* gene (six loci). In the red box plot diagrams, the horizontal line within each box represents the median; the limits of each box are the interquartile ranges, the whiskers are the maximum and minimum values, and the green horizontal bar within each box depicts the mean value. The numbers under the green horizontal bar denote the mean methylation score. The *P* values above the square panels are statistical differences among any two individual groups calculated by the Steel–Dwass test. **b** Representative immunohistochemical images for the cytotoxic T-cell marker CD8 (brown) in *POLE*-mutant CRC samples. **c** Association among age at diagnosis, *MLH1*-AB region methylation status, and the number of CD8-positive ( +) cells per HPF in *POLE*-mutant CRCs. The *P* value is calculated by the Kruskal–Wallis test. Red circles denote patients with *POLE* proofreading mutations. HPF; high-power field (× 400)

Using *MLH1*, *MGMT*, and *SFRP2* methylation status, we calculated the mean methylation score for each subgroup. When methylation data were analysed using the discrete regions in the promoter of the three genes, the mean methylation score was significantly higher in MSI-M, while the mean methylation score in POLE was the same as that in MSI-U and non-MSI (MSI-M: 5.2; POLE: 2.5; MSI-U: 2.2; non-MSI: 2.0, Fig. 5a).

Finally, Table 2 presents detailed information on the four *POLE*-mutant CRC patients. Somatic *POLE* mutations in CRCs are associated with enhanced tumour immunogenicity and increased CD8-positive lymphocytic infiltration [7, 13, 14, 21]. We examined CD8-positive infiltration in the four *POLE*-mutant CRC tissues (Fig. 5b). The number of CD8-positive cells per high-power field (HPF, × 400) was increased in *POLE*-mutant patients over 60 years of age at diagnosis, who possessed *MLH1*-AB region methylation (Table 2). For reference, we examined the association between age at diagnosis, *MLH1*-AB region methylation status, and the number of CD8-positive cells per HPF (Fig. 5c). The mean age at diagnosis of patients with unmethylation in the *MLH1-*AB region was significantly younger than that of patients with methylation in the *MLH1*-AB region (65.5 years old [range, 21–95] vs. 68.7 years old [range, 37–89] years, *P* = 0.001). Although tumour-infiltrating lymphocytes were likely to be more common in older *POLE*-mutant patients and/or with methylation in the *MLH1*-AB region, the analysed number of patients was too small to find associations.

**Table 2:** Detailed information of the four CRC patients with *POLE* proofreading mutations

ID	Sex	Age	*POLE* mutational spectrum	Tumour location	Histology	UICC stage						Recurrence	Methylation rate						CD8(/HPF)
													*MLH1*		*MGMT*		*SFRP2*		
							T	*N*	M	ly	v		AB	C	Mp	Eh	R1	R2	
123	F	63	S459F	Ascending colon	mod	IIA	3	0	0	0	0	No	45.1	0.0	0.0	0.0	43.7	35.8	110
777	F	61	P286R	Ascending colon	mod	IIA	3	0	0	0	0	No	74.1	0.0	0.0	0.0	61.9	45.6	39
945	M	45	P286R	Ascending colon	mod	IIA	3	0	0	0	1	No	0.0	0.0	0.0	0.0	91.8	69.9	25
596	M	41	V411L	Ascending colon	mod > muc	I	1	0	0	0	0	No	0.0	0.0	0.0	0.0	58.7	18.2	3

## Discussion

In the present study, we report a biologically distinct subset of CRCs with pathogenic somatic *POLE* mutations, especially those with simultaneous epigenetic alterations. As previously reported, pathogenic somatic *POLE* mutations are usually found in the early stages of CRC in the right side of the colon and relatively younger patients with CRC.

CIN, MSI, and CIMP constitute the three major mechanisms of genomic or epigenetic instability in CRC [1, 2, 5]. Experimental evidence has consistently supported the presence of CIMP in a subset of CRCs and correlates with the presence of *BRAF* V600E mutations [1, 22, 23]. In terms of CIMP development, it is debatable whether *BRAF* mutations can directly induce CIMP [24, 25]. Recently, Tao et al. [4] reported that the *BRAF* V600E mutation does not directly cause CIMP, and thus, aging-like acquisition of DNA methylation may favour the survival of cells with such mutations in the *BRAF* gene through suppression of senescence and activation of stem cell pathways.

Clinically, most sporadic MSI CRCs arise from a CIMP background caused by the epigenetic silencing of the *MLH1* gene, which justifies our reason for selecting methylation status of the *MLH1* gene at the beginning as a critical biomarker for classifying CRCs [1, 26]. CIN and CIMP have been proposed to represent two major mechanisms of genomic and epigenetic instability in CRC, and up to 50% of CRCs may be CIMP positive [1]. Indeed, CIMP determinations using CIMP-related markers have consistently identified clusters of CRCs with MSI and *BRAF* V600E mutations, but rarely *KRAS* mutations [27, 28]. However, when additional methylation loci are investigated, additional subsets of CRCs have been identified with extensive methylation; these tumours are non-MSI and are associated with mutations in the *KRAS* gene [1, 17, 29]. *BRAF* and *KRAS* gene products function in the same MAP-kinase signalling pathway, and activating mutations in these genes occur mutually exclusive [30, 31]. Interestingly, before the discovery of *BRAF* mutations, *KRAS* mutations have been proposed as a possible cause of aberrant methylation. Fibroblasts transformed by fos or ras show upregulated DNA methyltransferase expression and consequent global hypermethylation [32]. Indeed, as previously reported, most non-conventional CIMP markers are more likely to gain methylation in CRCs with *KRAS* mutations [26]. Therefore, we hypothesised that CIMP in CRC would result from activating mutations in either *BRAF* or *KRAS*. From this point of view, it might be sufficient to summarise the features of methylation by using the *MLH1*, *MGMT*, and *SFRP2* genes.

In the case of *KRAS* mutations, promoter methylation within the *MGMT* gene, which encodes a DNA repair gene that removes pro-mutagenic O^6^-methylguanine residues from DNA, is associated with *KRAS*-mutant CRC [17, 26, 33]. Thus, in this study, we examined *MGMT* methylation status to clarify the epigenetic features of *POLE*-mutant CRCs. Interestingly, although half of the *POLE*-mutant CRCs showed partial methylation in the *MLH1* promoter, none of the *POLE*-mutant CRCs displayed any methylation in the discrete promoter regions of the *MGMT* gene.

In addition to *MLH1* and *MGMT*, we evaluated *SFRP2* methylation (partial or extensive) because most CRCs possess extensive methylation in their promoter regions [34–36]. An interesting feature of *SFRP2* methylation is that extensive methylation is rare in adenomatous polyps, while it is common in CRCs [35, 36]. Additionally, extensive *SFRP2* methylation is more frequently observed in CRCs with *KRAS* mutations than in those with *BRAF* V600E mutations and those with wild-type *KRAS* or *BRAF* mutations [26, 35]. Thus, although extensive methylation in *MGMT* and *SFRP2* has a similar feature in terms of association with CRC with *KRAS* mutations, all *POLE*-mutant tumours demonstrated extensive methylation in *SFRP2*, but no methylation in *MGMT.*

Temko and colleagues demonstrated that acquisition of *POLE* mutations induces a distinct pattern of mutations in cancer driver genes, a substantially increased mutation burden, and an enhanced immune response that is detectable even in precancerous lesions [14]. Similar to this distinct genetic mutation pattern, although the mean methylation score in *POLE*-mutant tumours was similar to that in the MSI-U and non-MSI groups, *POLE* mutations may also cause a distinct pattern of epigenetic alterations in cancer-associated genes.

*POLE*-mutant CRCs have been reported to have a favourable prognosis, as noted for early-stage dMMR tumours [7]. The four *POLE*-mutant CRC patients in this study were diagnosed at stages I to II, and there was no recurrence within five years after surgical resection. IHC analysis showed that the number of CD8-positive cytotoxic tumour-infiltrating lymphocytes in *POLE*-mutant CRCs significantly increased in *POLE*-wild-type CRCs, especially in mismatch repair protein-proficient CRCs [7, 14]. We also examined the number of CD8-positive cells and compared them to the *MLH1*-AB region methylation status and age at diagnosis. In this study, CD8-positive cells were likely to be more common in older *POLE*-mutant patients and/or with methylation in the *MLH1*-AB region, but the number of analysed *POLE*-mutant patients was too small to reach statistical significance.

## Conclusions

Although we analysed over 1,000 CRC samples, CRCs with *POLE* proofreading mutations were found in only four tumours. Therefore, this rarity suggests that our results should be interpreted with caution. We conclude that CRC patients with *POLE* mutations are rare, such mutations are observed in younger individuals, lesions are often located within the right colon, diagnosis occurs at an earlier stage, and distinct epigenetic alterations might be associated with CD8 cell infiltration.

## Methods

### Aim of the study

This study aimed to clarify the incidence of *POLE* proofreading mutations in a large cohort of CRC patients in Japan and to evaluate the epigenetic profiles of *POLE*-mutant CRC and elucidate the clinicopathological features associated with key genetic and epigenetic alterations in this malignancy.

### Study participants and sample collection

A cohort of 1,052 CRC patients underwent surgical resection between 1998 and 2017 at the Okayama University Hospital, Japan. Tumour specimens and corresponding normal mucosa samples were collected according to institutional review board (IRB)-approved protocols (genome 270 and genome 271 at Okayama University; 3196–1 and 3239 at Kawasaki Medical School).

### Extraction of DNA and bisulphite conversion

Genomic DNA was extracted from fresh-frozen samples using a QIAamp DNA Mini Kit (Qiagen NV, Hilden, Netherlands). The extracted DNA was quantified using a Qubit 4 fluorometer with a Qubit dsDNA BR assay kit (Thermo Fisher Scientific, Cleveland, OH, USA). After determining the DNA quality, an EZ DNA methylation kit (Zymo Research, USA) was used for bisulfite conversion of the normalised samples.

### Detection of pathogenic POLE proofreading mutations

Pathogenic *POLE* hotspot mutations in the proofreading domain (exons 9, 13, and 14) were evaluated by Sanger sequencing. The primer sequences used are listed in Additional file 2: Table 2. PCR products were purified using a QIAquick PCR purification kit (Qiagen) and directly sequenced using an ABI PRISM® 3100-Avant Genetic Analyser (Applied Biosystems) and a SeqStudio Genetic Analyser (Thermo Fisher Scientific).

### MSI analysis

MSI status was analysed in all CRC tissues using four mononucleotide repeat markers (*BAT26*, *NR21*, *NR27*, and *CAT25*), as described previously [12, 16]. When at least one or more mononucleotide repeat markers displayed MSI, tumours were defined as having an MSI phenotype [37]. However, tumours without MSI in the four mononucleotide repeat markers were defined as having a non-MSI phenotype, as described in our previous studies [12, 16].

### MMR protein and CD8 immunohistochemistry

We employed immunohistochemistry to examine the MMR protein expression of MLH1, MSH2, PMS2, and MSH6 in primary tumour tissues of CRC specimens that showed the MSI phenotype. Staining was performed manually using FFPE specimens. Thin (5 µm) sections of representative blocks were deparaffinised and dehydrated using gradient solvents. Following antigen retrieval in citrate buffer (pH 6.0), endogenous peroxidase was blocked with 3% H_2_O_2_. Thereafter, the slides were incubated overnight in the presence of purified mouse monoclonal antibodies against MLH1 (clone G168-15, BD Pharmingen, San Diego, CA; dilution 1:50), MSH2 (clone G219-1129, BD Pharmingen, San Diego, CA; dilution 1:200), PMS2 (clone A16-4, BD Pharmingen San Diego, CA; dilution 1:200), and MSH6 protein (clone 44/MSH6, BD Pharmingen San Diego, CA; dilution 1:100). Further incubation was performed with a secondary antibody and the avidin–biotin–peroxidase complex (Vector Laboratories, Burlingame, CA, USA), followed by incubation with biotinyl tyramide and streptavidin–peroxidase. Diaminobenzidine was used as a chromogen, and haematoxylin was used as a nuclear counterstain. Tumour cells were scored negative for MMR protein expression only if the epithelial cells within the tumour tissue lacked nuclear staining, while the surrounding stromal cells showed positive staining. Samples showing proficiency in all MMR protein expressions were defined as pMMR, and samples showing deficiency in at least one of the four MMR proteins were defined as dMMR. When a tumour showed neither MLH1 nor PMS2 with staining, the tumour was classified as MLH1-deficient (dMLH1); when a tumour showed neither MSH2 nor MSH6 with staining, the tumour was classified as MSH2-deficient (dMSH2); when a tumour showed negative staining only for PMS2 but was positive for MLH1, the tumour was classified as PMS2-deficient (dPMS2), and when a tumour showed negative staining only for MSH6 but was positive for MSH2, the tumour was classified as MSH6-deficient (dMSH6).

IHC analysis for CD8 was performed in the four *POLE*-mutant tumours, as described previously [13]. The number of CD8-positive cells in the epithelial and stromal regions was quantified. The CD8 count per case was evaluated in a high-power field (HPF, × 400).

### DNA methylation detection within discrete regions of the MLH1, MGMT, and SFRP2 gene promoters

In addition to *MLH1*, we examined the discrete regions of *MGMT* and *SFRP2* promoters that affect these expressions or other critical features in tumorigenesis to clarify the epigenetic features in tumours with *POLE* mutations. Like *MLH1*, the *MGMT* gene is inactivated as a consequence of extensive methylation in its promoter region (dense methylation through minimal promoter [Mp] region to enhancer [Eh] region) [17, 26, 38, 39]. Extensive methylation in *MGMT* is significantly associated with MGMT protein downregulation and an increased burden of *KRAS* mutations in CRC patients [17, 33, 38]. Consistent with this epigenetic feature, dense methylation of regions 1 and 2 within the *SFRP2* gene promoter is a definitive feature of advanced CRCs [4, 34–36].

To quantify the population of methylated alleles of the *MLH1*,* MGMT*, and *SFRP2* promoters in each sample, a modified combined bisulfite restriction analysis (COBRA) with fluorescence dyes was performed to quantitatively measure the methylation density [12, 36, 38]. The primer sequences and restriction enzymes used are listed in Additional file 2: Table 2. PCR products digested with HhaI, RsaI, or Bst UI (New England BioLabs, Ipswich, MA, USA) were loaded simultaneously onto a SeqStudio Genetic Analyser (Thermo Fisher Scientific). Signals from individual PCR products were distinguished by the unique fluorescent PCR signal from each target and their fragment length, and the data were analysed using GeneMapper software 5 (Applied Biosystems, Foster City, CA, USA). In this study, the percentages of methylated CpG sites (digested by restriction enzymes) were calculated by determining the ratios between the restriction enzyme-cleaved PCR products and the total amount of PCR product in each locus, which was defined as the percentage of methylated CpG sites at 5.0% or more.

In our cohort of 1,052 CRCs, due to technical challenges in performing fluorescence bisulfite PCRs for the *MLH1*, *MGMT*, or *SFRP2* genes, two tumours that were otherwise characterised as dMLH1 by IHC analysis and 37 tumours without MSI were excluded from further analysis (Fig. 1).

### Detection of BRAF and KRAS mutations

Sanger sequencing was performed to confirm mutations in *KRAS* exon 2 and *BRAF* exon 15 (including codon 600), as described previously [12, 26]. PCR products were purified using a QIAquick PCR purification kit (Qiagen) and directly sequenced using an ABI PRISM® 3100-Avant Genetic Analyser (Applied Biosystems) and a SeqStudio Genetic Analyser (Thermo Fisher Scientific).

### Statistical analysis

All statistical analyses were performed using JMP Pro software (version 14.3; SAS Institute, Inc., Cary, NC, USA). Methylation levels in the *MLH1*, *MGMT*, and *SFRP2* promoters were analysed as both continuous and categorical variables (methylation level ≥ 5%; unmethylated: methylation level < 5%).

Categorical variables were compared using the Chi-squared test. The pair-wise comparisons for each subgroup were performed using a nonparametric multiple comparison method using the Steel–Dwass test. The association between age at dignosis and *MLH1*-AB region methylation status was evaluated by the Kruskal-Wallis test. All reported *P* values were two-sided, and *statistical significance was set at P* < 0.05.

## Supplementary Information

**Additional file 1**. Table 1. Detailed information of 65 MSI CRC patients.

**Additional file 2**. Table 2. Primer sequences.

## Data Availability

The datasets used and/or analysed in the current study are available from the corresponding author upon reasonable request.

## References

[1] Goel A, Nagasaka T, Arnold CN, Inoue T, Hamilton C, Niedzwiecki D, Compton C, Mayer RJ, Goldberg R, Bertagnolli MM (2007). The CpG island methylator phenotype and chromosomal instability are inversely correlated in sporadic colorectal cancer. Gastroenterology.

[2] Al-Sohaily S, Biankin A, Leong R, Kohonen-Corish M, Warusavitarne J (2012). Molecular pathways in colorectal cancer. J Gastroenterol Hepatol.

[3] Bogaert J, Prenen H (2014). Molecular genetics of colorectal cancer. Ann Gastroenterol.

[4] Tao Y, Kang B, Petkovich DA, Bhandari YR, In J, Stein-O'Brien G, Kong X, Xie W, Zachos N, Maegawa S (2019). Aging-like spontaneous epigenetic silencing facilitates wnt activation, stemness, and braf(V600E)-induced tumorigenesis. Cancer Cell.

[5] Issa JP, Shen L, Toyota M (2005). CIMP, at last. Gastroenterology.

[6] Cancer Genome Atlas N: Comprehensive molecular characterization of human colon and rectal cancer. Nature 2012;487(7407):330–7. 10.1038/nature11252 PMC3401966 22810696

[7] Domingo E, Freeman-Mills L, Rayner E, Glaire M, Briggs S, Vermeulen L, Fessler E, Medema JP, Boot A, Morreau H (2016). Somatic POLE proofreading domain mutation, immune response, and prognosis in colorectal cancer: a retrospective, pooled biomarker study. Lancet Gastroenterol Hepatol.

[8] Hino H, Shiomi A, Kusuhara M, Kagawa H, Yamakawa Y, Hatakeyama K, Kawabata T, Oishi T, Urakami K, Nagashima T (2019). Clinicopathological and mutational analyses of colorectal cancer with mutations in the POLE gene. Cancer Med.

[9] Guerra J, Pinto C, Pinto D, Pinheiro M, Silva R, Peixoto A, Rocha P, Veiga I, Santos C, Santos R (2017). POLE somatic mutations in advanced colorectal cancer. Cancer Med.

[10] Cancer Genome Atlas Research N, Kandoth C, Schultz N, Cherniack AD, Akbani R, Liu Y, Shen H, Robertson AG, Pashtan I, Shen R *et al*: Integrated genomic characterization of endometrial carcinoma. Nature 2013, 497(7447):67–73. 10.1038/nature12113 PMC3704730 23636398

[11] Rayner E, van Gool IC, Palles C, Kearsey SE, Bosse T, Tomlinson I, Church DN (2016). A panoply of errors: polymerase proofreading domain mutations in cancer. Nat Rev Cancer.

[12] Haruma T, Nagasaka T, Nakamura K, Haraga J, Nyuya A, Nishida T, Goel A, Masuyama H, Hiramatsu Y (2018). Clinical impact of endometrial cancer stratified by genetic mutational profiles, POLE mutation, and microsatellite instability. PLoS ONE.

[13] van Gool IC, Eggink FA, Freeman-Mills L, Stelloo E, Marchi E, de Bruyn M, Palles C, Nout RA, de Kroon CD, Osse EM (2015). POLE proofreading mutations elicit an antitumor immune response in endometrial cancer. Clin Cancer Res.

[14] Temko D, Van Gool IC, Rayner E, Glaire M, Makino S, Brown M, Chegwidden L, Palles C, Depreeuw J, Beggs A (2018). Somatic POLE exonuclease domain mutations are early events in sporadic endometrial and colorectal carcinogenesis, determining driver mutational landscape, clonal neoantigen burden and immune response. J Pathol.

[15] Palles C, Cazier JB, Howarth KM, Domingo E, Jones AM, Broderick P, Kemp Z, Spain SL, Guarino E, Salguero I (2013). Germline mutations affecting the proofreading domains of POLE and POLD1 predispose to colorectal adenomas and carcinomas. Nat Genet.

[16] Takehara Y, Nagasaka T, Nyuya A, Haruma T, Haraga J, Mori Y, Nakamura K, Fujiwara T, Boland CR, Goel A (2018). Accuracy of four mononucleotide-repeat markers for the identification of DNA mismatch-repair deficiency in solid tumors. J Transl Med.

[17] Nagasaka T, Sasamoto H, Notohara K, Cullings HM, Takeda M, Kimura K, Kambara T, MacPhee DG, Young J, Leggett BA (2004). Colorectal cancer with mutation in BRAF, KRAS, and wild-type with respect to both oncogenes showing different patterns of DNA methylation. J Clin Oncol Off J Am Soc Clin Oncol.

[18] Deng G, Chen A, Hong J, Chae HS, Kim YS (1999). Methylation of CpG in a small region of the hMLH1 promoter invariably correlates with the absence of gene expression. Cancer Res.

[19] Miyakura Y, Sugano K, Konishi F, Ichikawa A, Maekawa M, Shitoh K, Igarashi S, Kotake K, Koyama Y, Nagai H (2001). Extensive methylation of hMLH1 promoter region predominates in proximal colon cancer with microsatellite instability. Gastroenterology.

[20] Nagasaka T, Goel A, Matsubara N, Tanaka N (2006). Detection of fecal DNA methylation for colorectal neoplasia: does it lead to an optimal screening test?. Acta Med Okayama.

[21] Howitt BE, Shukla SA, Sholl LM, Ritterhouse LL, Watkins JC, Rodig S, Stover E, Strickland KC, D'Andrea AD, Wu CJ (2015). Association of polymerase e-mutated and microsatellite-instable endometrial cancers with neoantigen load, number of tumor-infiltrating lymphocytes, and expression of PD-1 and PD-L1. JAMA Oncol.

[22] Ogino S, Kawasaki T, Kirkner GJ, Loda M, Fuchs CS (2006). CpG island methylator phenotype-low (CIMP-low) in colorectal cancer: possible associations with male sex and KRAS mutations. J Mol Diagn.

[23] Yamamoto E, Suzuki H, Yamano HO, Maruyama R, Nojima M, Kamimae S, Sawada T, Ashida M, Yoshikawa K, Kimura T (2012). Molecular dissection of premalignant colorectal lesions reveals early onset of the CpG island methylator phenotype. Am J Pathol.

[24] Feng Y, Sentani K, Wiese A, Sands E, Green M, Bommer GT, Cho KR, Fearon ER (2013). Sox9 induction, ectopic Paneth cells, and mitotic spindle axis defects in mouse colon adenomatous epithelium arising from conditional biallelic Apc inactivation. Am J Pathol.

[25] Hinoue T, Weisenberger DJ, Lange CP, Shen H, Byun HM, Van Den Berg D, Malik S, Pan F, Noushmehr H, van Dijk CM (2012). Genome-scale analysis of aberrant DNA methylation in colorectal cancer. Genome Res.

[26] Nagasaka T, Koi M, Kloor M, Gebert J, Vilkin A, Nishida N, Shin SK, Sasamoto H, Tanaka N, Matsubara N (2008). Mutations in both KRAS and BRAF may contribute to the methylator phenotype in colon cancer. Gastroenterology.

[27] Samowitz WS, Albertsen H, Herrick J, Levin TR, Sweeney C, Murtaugh MA, Wolff RK, Slattery ML (2005). Evaluation of a large, population-based sample supports a CpG island methylator phenotype in colon cancer. Gastroenterology.

[28] Ogino S, Kawasaki T, Kirkner GJ, Kraft P, Loda M, Fuchs CS (2007). Evaluation of markers for CpG island methylator phenotype (CIMP) in colorectal cancer by a large population-based sample. J Mol Diagn.

[29] Iacopetta B, Grieu F, Li W, Ruszkiewicz A, Caruso M, Moore J, Watanabe G, Kawakami K (2006). APC gene methylation is inversely correlated with features of the CpG island methylator phenotype in colorectal cancer. Int J Cancer Journal international du cancer.

[30] Davies H, Bignell GR, Cox C, Stephens P, Edkins S, Clegg S, Teague J, Woffendin H, Garnett MJ, Bottomley W (2002). Mutations of the BRAF gene in human cancer. Nature.

[31] Kim JS, Lee C, Foxworth A, Waldman T (2004). B-Raf is dispensable for K-Ras-mediated oncogenesis in human cancer cells. Can Res.

[32] Ordway JM, Williams K, Curran T (2004). Transcription repression in oncogenic transformation: common targets of epigenetic repression in cells transformed by Fos, Ras or Dnmt1. Oncogene.

[33] Esteller M, Toyota M, Sanchez-Cespedes M, Capella G, Peinado MA, Watkins DN, Issa JP, Sidransky D, Baylin SB, Herman JG (2000). Inactivation of the DNA repair gene O6-methylguanine-DNA methyltransferase by promoter hypermethylation is associated with G to A mutations in K-ras in colorectal tumorigenesis. Cancer Res.

[34] Suzuki H, Watkins DN, Jair KW, Schuebel KE, Markowitz SD, Chen WD, Pretlow TP, Yang B, Akiyama Y, Van Engeland M (2004). Epigenetic inactivation of SFRP genes allows constitutive WNT signaling in colorectal cancer. Nat Genet.

[35] Takeda M, Nagasaka T, Dong-Sheng S, Nishie H, Oka T, Yamada E, Mori Y, Shigeyasu K, Morikawa T, Mizobuchi S (2011). Expansion of CpG methylation in the SFRP2 promoter region during colorectal tumorigenesis. Acta Med Okayama.

[36] Nagasaka T, Tanaka N, Cullings HM, Sun DS, Sasamoto H, Uchida T, Koi M, Nishida N, Naomoto Y, Boland CR (2009). Analysis of fecal DNA methylation to detect gastrointestinal neoplasia. J Natl Cancer Inst.

[37] Goel A, Nagasaka T, Hamelin R, Boland CR (2010). An optimized pentaplex PCR for detecting DNA mismatch repair-deficient colorectal cancers. PLoS ONE.

[38] Nagasaka T, Goel A, Notohara K, Takahata T, Sasamoto H, Uchida T, Nishida N, Tanaka N, Boland CR, Matsubara N (2008). Methylation pattern of the O6-methylguanine-DNA methyltransferase gene in colon during progressive colorectal tumorigenesis. Int J Cancer Journal international du cancer.

[39] Pegg AE (1990). Mammalian O6-alkylguanine-DNA alkyltransferase: regulation and importance in response to alkylating carcinogenic and therapeutic agents. Can Res.

